# Quantitative study of the changes in brain white matter before and after radiotherapy by applying multi-sequence MR radiomics

**DOI:** 10.1186/s12880-022-00816-3

**Published:** 2022-05-13

**Authors:** Mingming Chen, Lizhen Wang, Guanzhong Gong, Yong Yin, Pengcheng Wang

**Affiliations:** 1 College of Radiology, Shandong First Medical University & Shandong Academy of Medical Sciences, 250117 Jinan, China; 2 Department of Radiation Physics, Shandong First Medical University Affiliated Cancer Hospital, Shandong Cancer Hospital and Institute (Shandong Cancer Hospital), 250117 Jinan, China

**Keywords:** Radiomics, Brain white matter, Radiotherapy, Multisequence MR, Dose gradients

## Abstract

**Purpose:**

To analyse the changes in brain white matter before and after radiotherapy (RT) by applying multisequence MR radiomics features and to establish a relationship between the changes in radiomics features and radiation dose.

**Methods:**

Eighty-eight patients with brain tumours who had undergone RT were selected in this study, and MR images (T1, T1+C, T2FLAIR, T2, DWI, and ASL) before and after RT were obtained. The brain white matter was delineated as an ROI under dose gradients of 0–5 Gy, 5–10 Gy, 10–15 Gy, 15–20 Gy, 20–30 Gy, 30–40 Gy, and 40–50 Gy. The radiomics features of each ROI were extracted, and the changes in radiomics features before and after RT for different sequences under different dose gradients were compared.

**Results:**

At each dose gradient, statistically significant features of different MR sequences were mainly concentrated in three dose gradients, 5–10 Gy, 20–30 Gy, and 30–40 Gy. The T1+C sequence held the most features (66) under the 20–30 Gy dose gradient. There were 20 general features at dose gradients of 20–30 Gy, 30–40 Gy, and 40–50 Gy, and the changes in features first decreased and then increased following dose escalation. With dose gradients of 5–10 Gy and 10–15 Gy, only T1 and T2FLAIR had general features, and the rates of change were − 24.57% and − 29.32% for T1 and − 3.08% and − 10.87% for T2FLAIR, respectively. The changes showed an upward trend with increasing doses. For different MR sequences that were analysed under the same dose gradient, all sequences with 5–10 Gy, 20–30 Gy and 30–40 Gy had general features, except the T2FLAIR sequence, which was concentrated in the FirstOrder category feature, and the changes in features of T1 and T1+C were more significant than those of the other sequences.

**Conclusions:**

MR radiomics features revealed microscopic changes in brain white matter before and after RT, although there was no constant dose-effect relationship for each feature. The changes in radiomics features in different sequences could reveal the radiation response of brain white matter to different doses.

## Introduction

Radiotherapy (RT) has become one of the main treatments for brain tumours. Patients receiving RT can achieve long-term survival, although most of these patients may have a certain degree of radiation-induced brain injury (RBI) [1]. With the development of imaging and RT technology, the accuracy and conformability of RT have been significantly improved. However, RBI continues to be an obstacle that affects treatment and the quality of life of patients. Therefore, the early accurate prediction and diagnosis of RBI are vital [2]. RBI can be categorized as acute (several days to several weeks after RT), early-delayed (1–6 months after RT), and late-delayed effects (more than 6 months after RT, usually irreversible). Diffuse white matter injury is one of the characteristics of late RBI [3].

The development of RBI is directly related to the irradiated volume and radiation dose. Radiation dose is one of the main factors causing RBI. Animal models of RBI have also demonstrated that radiation injury is directly related to the irradiated volume [4]. Most studies have focused on the influence of RT on the hippocampal network and memory decline. With the development of radiobiology, people have realized that brain injury is not limited to the hippocampus and memory but also includes white matter injuries [5].

Brain white matter is more sensitive to radiation because of its abundant blood supply, which in turn leads to cognitive and memory disorders and seriously affects the quality of life of patients [6]. From a radiobiology perspective, brain white matter injury will have a series of biological effects with changes in the radiation dose. Presently, the diagnosis of white matter injury mainly depends on magnetic resonance imaging (MRI). However, conventional anatomic MR images cannot objectively show the early injury effect, and traditional methods of predicting injury based on the dose-volume index are unable to dynamically track the dose of brain tissue during RT, which leads to an inability or delay in the early prediction of injury. Recently, diffusion tensor imaging (DTI) has been used in studies based on white matter injury. However, the spatial resolution of DTI limits the analysis of main white matter bundles, which makes it difficult to reveal subtle changes in regional white matter structure [7, 8]. The rapidly developing radiomics achieved by mining a large amount of in-depth information in the images could be used to quantitatively analyse the dynamic changes in tumour or normal tissue during treatment, which may help reveal and track the microscopic changes of early white matter injury [9].

As the MR radiomics features of different sequences could reflect different information, in this study, multisequence MRI radiomics was used to investigate the changes in the white matter before and after RT under different dose gradients and to analyse the correlation between the changes in radiomics features and dose gradients to provide an objective basis for the early prediction, diagnosis, and dynamic tracking of RBI.

## Materials and methods

### Patient information

A total of 88 patients with brain tumours who had received RT at the Shandong Cancer Hospital from September 2018 to May 2021 were analysed retrospectively. Among them, 24 patients received whole-brain RT, and 64 patients received local RT. There were 47 males with an average age of 52.9 years and 41 females with an average age of 50.5 years. The tumour types were divided into six categories (56 cases of glioma, 25 cases of brain metastases, 2 cases of brain lymphoma, 3 cases of meningioma, 1 case of fibrosarcoma, and 1 case of ependymoma).

## Methods

### CT simulation

The heads of all patients were fixed with a thermoplastic film, and the scan was achieved by Philips Brilliance Big Bore CT (Philips, Netherlands). The patients were placed in the supine position, and CT scans were performed with 3 mm slice thickness and 3 mm slice gap.

### MR Simulation

**Fig. 1:**
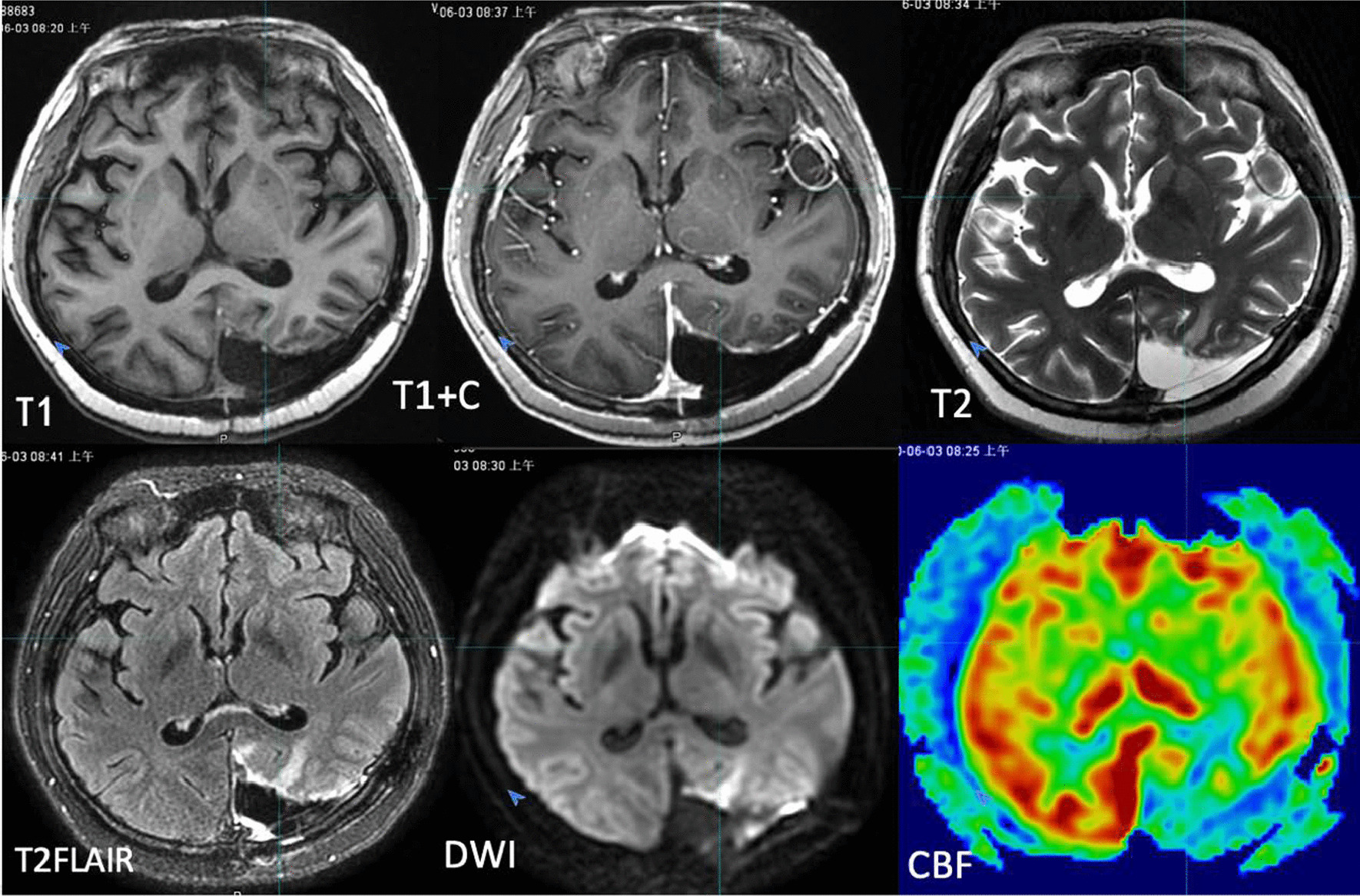
Schematic diagram of multisequence MRI of a patient. **A** T1; **B** T1+C; **C** T2FLAIR; **D** T2Propeller; **E** DWI; **F** CBF

After the completion of CT simulation, all the patients were scanned with MR in the same posture and fixed mode, and MR simulation images were obtained using a GE 3.0T superconducting MR scanner (Discovery 750w, GE Healthcare, USA) with six channels of head coils. The MR positioning sequences were (1) 3D T1WI (defined as T1); (2) 3D T1WI enhanced scanning (defined as T1+C); (3) T2 fluid-attenuated inversion recovery sequence (T2FLAIR); (4) T2 PROPELLOR; (5) diffusion-weighted imaging (DWI) with a B value of 1000 s/mm^2^; (6) and three-dimensional arterial spin labelling (3D-ASL) perfusion imaging of cerebral blood flow (CBF) (Fig. 1).

The contrast agent of the T1+C sequence adopted the paramagnetic contrast agent Gd-DTPA, the dose was 0.2 mL/kg, and the injection rate was 2 mL/s. The scan was completed within 3–5 min after the injection. Within 3–5 days after RT, MR scanning in the same body position and the same sequence was performed again, and the scanning parameters of different MR sequences are presented in Table 1.

**Table 1:** Scan parameters of different MR sequences

Parameter	Sequence					
	T1	T1+C	T2FLAIR	T2Propeller	DWI	CBF
TR (ms)	8.5	8.5	11,000	7019	15,293	5160
TE (ms)	3.2	3.2	124	114	97.4	11.5
FOV (cm)	25.6 × 25.6	25.6 × 25.6	26 × 26	26 × 26	26 × 26	25.6 × 25.6
Matrix (mm)	256 × 256	256 × 256	320 × 256	416 × 416	128 × 128	256 × 256
Thickness (mm)	3	3	3	3	3	3
Layer spacing (mm)	0	0	0	0	0	0

### Target delineation and planning design

The simulated CT and MR images were transmitted to the planning system Varian Eclipse (version 15.5, USA), in which the tumour target and organs at risk (OARs) were delineated and the RT plan was defined. Patients with local RT were treated with intensity-modulated radiotherapy (IMRT) at a prescribed dose of 2.0 Gy/fx25f, while those with whole-brain RT were treated with three-dimensional conformal radiotherapy (3D-CRT) at a prescribed dose of 2.0 Gy/fx20f. All patients received a single dose of 2.0 Gy.

### Regions of interest definition and CT/MR fusion

**Fig. 2:**
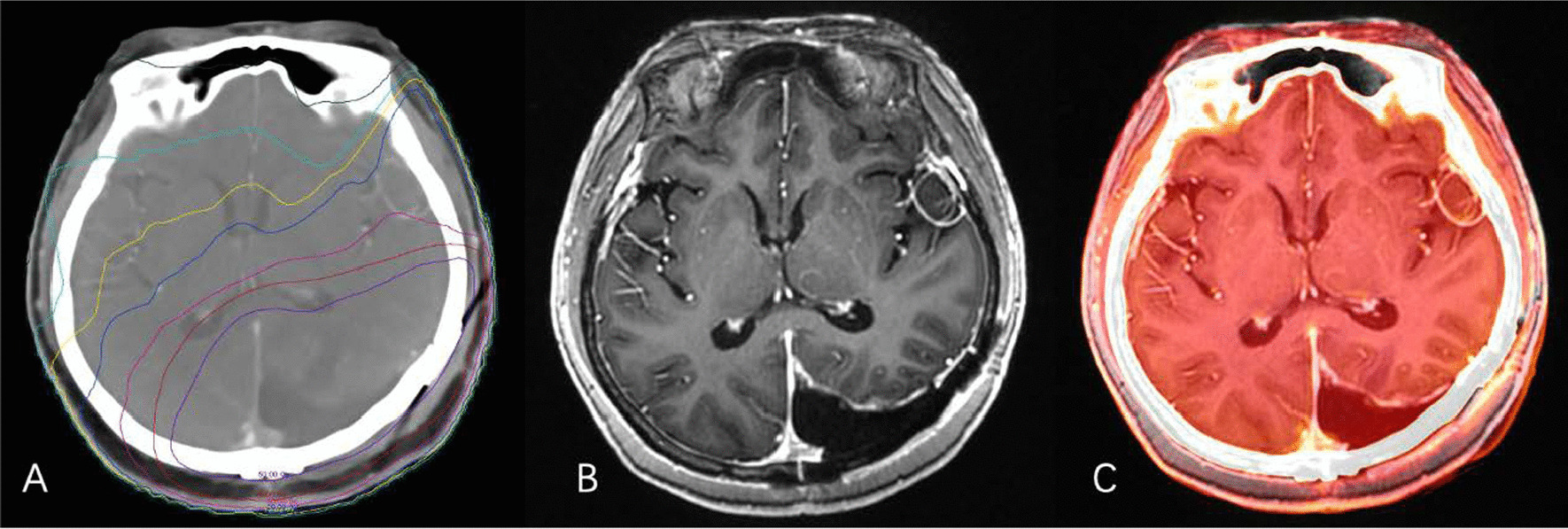
Registration of image fusion. **A**: Radiation dose distribution on a CT image; **B**: MR T1+C image; **C**: Fusion of the registered images

On the Varian Eclipse planning system, according to the actual irradiation dose of patients, the distribution maps of the doses 0–5 Gy, 5–10 Gy, 10–15 Gy, 15–20 Gy, 20–30 Gy, 30–40 Gy, and 40–50 Gy were obtained. The RT plan and MR images were imported into the software MIM Maestro (version 6.8.2, USA), and then the CT images were rigidly registered with MR images of different sequences before and after RT (Fig. 2).

Within the fusion dose line, a certain volume of white matter regions was delineated as regions of interest (ROI) on the seven dose gradient images of different MR sequences. The volume of a single ROI of patients with local RT was ≥ 0.5 cm^3^, while that of patients with whole-brain RT was ≥ 1 cm^3^. To ensure the consistency of the ROI position, the MR images of each sequence were rigidly registered, and then the local mismatch region was adjusted manually. When determining the ROI close to the high dose area, refer to the patient’s RT plan to exclude ROI and tumour PTV and keep the distance between them above 3 mm.

### Extraction of radiomics features

The software 3D Slicer (version 4.13.0, USA) was used to extract the radiomics features of each ROI. Before feature extraction, we use a fixed bin width of 25 to discretize the images. Then, each ROI yielded 93 features. Eighteen first-order features derived from the tumour intensity histogram reflected the distribution of the values of individual voxels without concern for spatial relationships. Seventy-five texture features described the spatial arrangement of voxels, as calculated from different parent matrices, which included the grey level dependence matrix (GLDM), the grey level cooccurrence matrix (GLCM), the grey level size zone matrix (GLSZM), the grey level run length matrix (GLRLM) and the neighbourhood grey-tone difference matrix (NGTDM). We used IBM SPSS (version 26.0, USA) to screen out statistically significant differences before and after RT. The data were analysed in two ways: (1) the changes in radiomics features of six sequences under different dose gradients before and after RT were compared, and their relationship with RT dose was studied. (2) The changes in radiomics features of different sequences under the same dose gradient before and after RT were compared.

### Statistical analysis

A paired t test was conducted using the statistical program IBM SPSS Statistics (version 26.0, USA), and *P* < 0.05 indicated statistically significant differences.

## Results

### The overlook of radiomics features for different MR sequences

The distribution of radiomics features with statistically significant differences under different MR sequences and different dose gradients was uneven, being mainly concentrated in the three dose gradients 5–10 Gy, 20–30 Gy, and 30–40 Gy, whereas only T1+C showed 34 features under the high-dose gradient of > 40 Gy (Table 2).

**Table 2:** Distribution of features with statistically significant differences under different sequences with different dose gradients

MR sequences	Dose gradient						
	0–5 Gy	5–10 Gy	10–15 Gy	15–20 Gy	20–30 Gy	30–40 Gy	40–50 Gy
T1	1	14	1	1	64	19	2
T1+C	1	2	1	0	66	55	34
T2FLAIR	0	5	2	1	0	1	0
T2PROPELLER	0	47	0	0	24	30	0
DWI	1	9	4	2	1	9	1
CBF	1	5	1	1	16	6	4

## Analysis of the changes in radiomics features of different dose gradients under the same sequence

### Comparative analysis of the radiomics features on T1 before and after RT

In T1, under dose gradients of 0–5 Gy, 5–10 Gy, 10–15 Gy, 15–20 Gy, 20–30 Gy, 30–40 Gy, and 40–50 Gy, there were 1, 14, 1, 1, 64, 19, and 2 radiomics features, which showed statistically significant differences before and after RT. Among these, the radiomics features were distributed more with 5–10 Gy, 20–30 Gy, and 30–40 Gy, which were concentrated in the FirstOrder and GLCM categories (Table 3).

**Table 3:** Number of features with statistically significant differences

Dose gradient	Radiomics feature						Total
	FirstOrder	GLCM	GLDM	GLRLM	GLSZM	NGTDM	
0–5 Gy	0	1	0	0	0	0	1
5–10 Gy	10	0	1	1	2	0	14
10–15 Gy	1	0	0	0	0	0	1
15–20 Gy	0	0	1	0	0	0	1
20–30 Gy	16	20	9	9	7	3	64
30–40 Gy	10	2	2	2	3	0	19
40–50 Gy	0	2	0	0	0	0	2

**Fig. 3:**
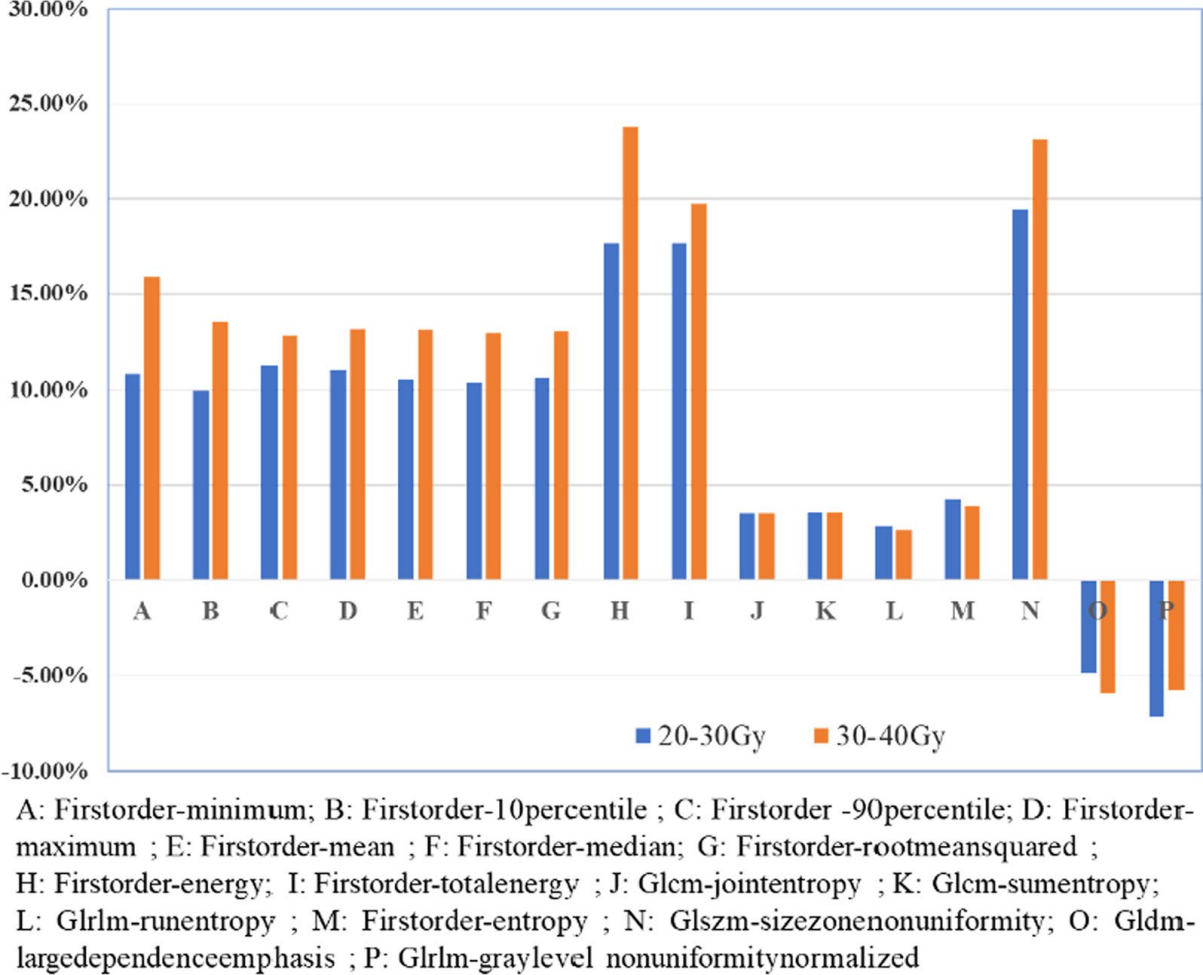
The rate of change analysis of general features on T1

Among these features, first-order skewness was a general feature under dose gradients of 5–10 Gy and 10–15 Gy; the rates of change were − 24.57 and − 29.32%, respectively, and the change showed an upwards trend with increasing dose. There were 16 general features under the gradient 20–30 Gy and 30–40 Gy, among which the features GLRLM-RunEntropy, FirstOrder-Entropy, and GLRLM-GreyLevel-NonUniformityNormalized showed a downwards trend with an increase in the dose. The rates of change of the other features increased with increasing dose (Fig. 3)

The radiomics features with the most changes before and after RT under different dose gradients were GLCM-Idn (-0.87%), FirstOrder-Skewness (-24.57%), FirstOrder-Skewness (-29.32%), GLDM-LargeDependenceHighGrayLevelEmphasis (8.41%), GLCM-ClusterProminence (36.23%), and FirstOrder-Energy (23.80%), among which the maximum changes in radiomics features were in FirstOrder-Skewness (5–10 Gy), GLCM-ClusterProminence (20–30 Gy), and FirstOrder-TotalEnergy (30–40 Gy).

### Comparative analysis of radiomics features on T1+C before and after RT

Under the seven dose gradients, there were 1, 2, 1, 0, 66, 55, and 34 radiomics features with statistically significant differences before and after RT, which, similar to T1, were mainly concentrated in 20–30 Gy, 30–40 Gy, and 40–50 Gy. Most were observed in the FirstOrder and GLCM category features (Table 4).

**Table 4:** The number of features with statistically significant differences on T1+C

Dose gradient	Radiomics feature						Total
	FirstOrder	GLCM	GLDM	GLRLM	GLSZM	NGTDM	
0–5 Gy	0	0	0	0	1	0	1
5–10 Gy	2	0	0	0	0	0	2
10–15 Gy	0	0	0	0	1	0	1
15–20 Gy	0	0	0	0	0	0	0
20–30 Gy	18	18	8	10	10	2	66
30–40 Gy	11	12	7	8	10	2	55
40–50 Gy	7	17	2	3	3	2	34

Among these features, the radiomics features with the most changes before and after RT under the dose gradients 0–5 Gy, 5–10 Gy, 10–15 Gy, 20–30 Gy, 30–40 Gy, and 40–50 Gy were GLSZM-GreyLevelNonUniformity (9.13%), FirstOrder-TotalEnergy (23.95%), GLSZM-LargeAreaHighGrayLevelEmphasis (−7.74%), FirstOrder-Skewness (−43.13%), GLSZM-SizeZoneNonUniformity (32.15%), and NGTDM-Complexity (49.21%), respectively

**Fig. 4:**
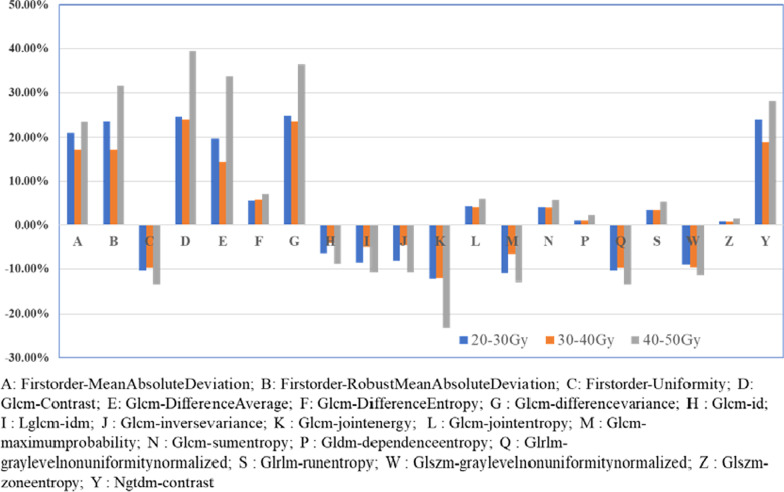
The rate of change analysis of general features on T1+C

There were 20 general features under dose gradients of 20–30 Gy, 30–40 Gy, and 40–50 Gy, which mainly included FirstOrder (3), GLCM (11), GLDM (1), GLSZM (2), GLRLM (2), and NGTDM (1). Among these, the changes in the feature GLSZM-GreyLevelNonUniformityNomalized under the dose gradients 20–30 Gy and 30–40 Gy showed a downwards trend with an increase in the dose, whereas the other features decreased at first and then increased in a dose-dependent manner (Fig. 4)

### Comparative analysis of the radiomics features on T2FLAIR before and after RT

Under different dose gradients of this sequence, there were 0, 5, 2, 1, 0, 1, and 0 radiomics features with statistically significant differences before and after RT. These numbers were significantly less than T1 and T1+C sequences, and there were no statistically significant differences in the dose gradients of 0–5 Gy (low-dose) and > 40 Gy (high-dose). Among these features, GLSZM-LargeAreaHighGrayLevelEmphasis under dose gradients of 5–10 Gy and 10–15 Gy was a general feature, with change rates of − 3.08% and − 10.87%, respectively; the change showed an upward trend with increasing dose.

### Comparative analysis of radiomics features on T2PROPELLER before and after RT

**Fig. 5:**
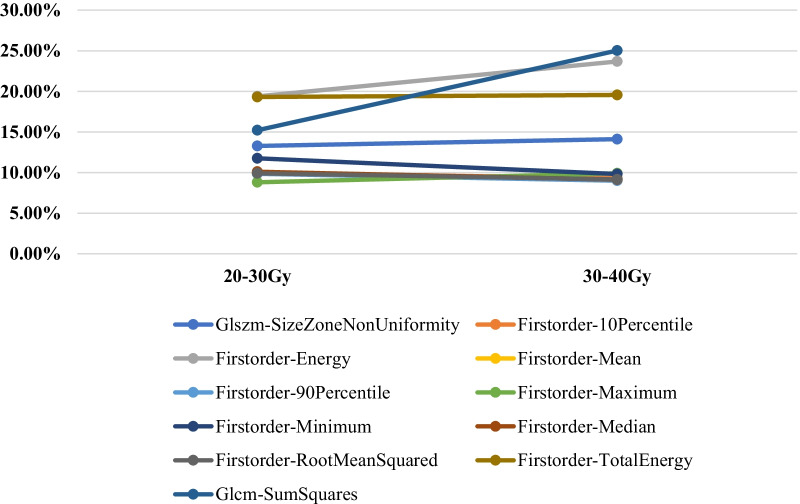
The rate of change analysis of general features on T2PROPELLER

Under different dose gradients of this sequence, there were 0, 47, 0, 0, 24, 30, and 0 radiomics features with statistically significant differences before and after RT, respectively. Similar to T2FLAIR, there was no significant change at dose gradients of 0–5 Gy and > 40 Gy. Among these features, there were significant changes in the radiomics features NGTDM-Contrast (32.43%), GLCM-ClusterTendency (20.22%), and GLDM-GreyLevelVariance (28.72%) only under dose gradients of 5–10 Gy, 20–30 Gy, and 30–40 Gy, respectively. There were 11 general features under the dose gradients of 20–30 Gy and 30–40 Gy, among which the features GLCM-SumSquares and FirstOrder-Energy showed an upwards trend with increasing dose, whereas the other features showed a negligible change (Fig. 5).

### Comparative analysis of the radiomics features on CBF before and after RT

Under seven dose gradients of this sequence, there were 1, 5, 1, 1, 16, 6, and 4 features with statistically significant differences before and after RT. Among these, the number of features was the largest under the 20–30 Gy gradient. The statistically significant differences before and after RT could be screened under all dose gradients, although there were no general features. Among these radiomics features, the most changes before and after RT were observed in GLCM-MCC (−16.41%), GLRLM-LongRunLowGrayLevelEmphasis (28.06%), GLCM-Imc1 (−15.06%), GLRLM-RunEntropy (−3.31%), GLSZM-SmallAreaEmphasis (−32.38%), GLSZM-LargeAreaHighGrayLevelEmphasis (33.25%), and GLDM-DependenceVariance (28.19%) in the dose gradient of 30–40 Gy (Table 5).

**Table 5:** Analysis of the rate of change in radiomics features of CBF

Dose gradient	The interval of feature change rate with statistically significant difference	The most significant feature of change
0–5 Gy	−16.41%	Without
5–10 Gy	−16.32–28.06%	GLRLM-LongRunLowGrayLevelEmphasis
10–15 Gy	−15.06%	Without
15–20 Gy	−3.31%	Without
20–30 Gy	−32.38–32.34%	GLSZM-SmallAreaEmphasis
30–40 Gy	−23.59–33.25%	GLSZM-LargeAreaHighGrayLvelEmphasis
40–50 Gy	−20.69–28.19%	GLDM-DependenceVariance

### Comparative analysis of radiomics features on DWI before and after RT

Under different dose gradients, the distribution of DWI image radiomics features with statistically significant differences before and after RT was relatively stable, with 1, 9, 4, 2, 1, 9, and 1 features in each dose gradient. There were no general features in all dose gradients, but there was a general feature, namely, FirstOrder-Median, under the dose gradients 20–30 Gy and 30–40 Gy, and its change showed an upwards trend with increasing dose. Additionally, there was a general feature GLSZM-GreyLevelVariance under the dose gradients 5–10 Gy and 10–15 Gy, with rates of change of 26.69% and 28.75%, respectively, and the change showed an upwards trend with an increase in the dose. Among these features, the radiomics features with the most changes under different dose gradients were GLCM-Correlation (−15.14%), FirstOrder-Skewness (−33.22%), GLSZM-GreyLevelVariance (28.75%), GLCM MaximumProbability (−6.07%), FirstOrder-Median (8.83%%), FirstOrder Minimum (13.56%), and GLCM-InverseVariance (−8.55%). In general, the general features were not screened under seven different dose gradients, although the changes in general features under individual dose gradients showed an upwards trend with increasing dose (Table 6).

**Table 6:** Analysis of the rate of change in radiomics features of DWI images

Dose gradient	The interval of feature change rate with statistically significant difference	The most significant feature of change
0–5 Gy	−15.14%	Without
5–10 Gy	−33.22–26.83%	Firstorder-Skewness
10–15 Gy	15.08–28.75%	GLSZM-GreyLevelVariance
15–20 Gy	−6.07–5.27%	Without
20–30 Gy	8.83%	Without
30–40 Gy	11.52–13.56%	Without
40–50 Gy	−8.55%	Without

## Analysis of radiomics features of different MR sequences under the same dose gradient

Under dose gradients of 0–5 Gy, 10–15 Gy, 15–20 Gy, and 40–50 Gy, few features were screened out by all MR sequences, and there were no general features that could cover more than four MR sequences. However, under the dose gradient of 30–40 Gy, all different MR sequences were significantly different, and six general features could be screened out by T1, T1+C, T2Propeller, and DWI sequences, with the most significant changes in the T1 and T1+ sequences (Table 7).

**Table 7:** Analysis of the rate of change of radiomics features of different MR sequences

Radiomics feature	MR sequence			
	T1 (%)	T1+C (%)	T2Propeller (%)	DWI (%)
Firstorder-minimum	15.86	15.20	9.82	13.56
Firstorder-maximum	13.16	13.90	9.91	11.76
Firstorder-mean	13.07	12.83	9.14	11.60
Firstorder-median	12.94	12.65	9.13	11.52
Firstorder-RootMeanSquared	13.04	12.82	9.16	11.59
Firstorder-TotalEnergy	19.74	20.31	19.56	11.85

### Analysis of changes in radiomics features under the dose gradient 20–30 gy

Under this dose gradient, the general feature FirstOrder-Median was screened out from T1, T1+C , T2Propeller, CBF, and DWI, and the rates of change were 10.40%, 14.23%, 10.10%, − 11.29%, and 8.83%, respectively. Based on the rates of change of general features, it was observed that the changes in the five sequences had little difference and tended to be stable under this dose gradient.

### Analysis of changes in radiomics features under the dose gradient 5–10 gy

FirstOrder-Energy and FirstOrder-TotalEnergy were selected as general features from T1, T1+C, T2Propeller, CBF, and DWI under the 5–10 Gy dose gradient, and the rates of change were T1 (16.14%, 20.80%), T1+C (23.95%, 23.95%), T2Propeller (23.05%, 22.34%), CBF (−15.06%, − 16.32%), and DWI (16.63%, 16.63%). Thus, the changes in T1 and T2 and CBF and DWI were similar, while the changes in the other sequence features except T2FLAIR were statistically significant under this gradient.

T2FLAIR could screen out relatively few meaningful features under all dose gradients. On the other hand, the general features in the T1, T1+C, T2Propeller, and DWI sequences could be screened under different dose gradients, and from the screened general features, the general features under different gradients and sequences were all FirstOrder category features, which indicated that these features tended to be stable under each MR sequence.

## Discussion

With the increasing application of RT in the comprehensive treatment of brain tumours, the incidence of RBI, mainly manifested as fatigue, somnolence, headache and nausea caused by cerebral oedema, delayed recall, cognitive impairment, and other adverse symptoms, is increasing and seriously affects the quality of life of patients [10]. The late stage of RBI involves radionecrosis, which includes symptoms such as pseudoprogression; thus, differentiating between radiation necrosis and progressive tumours remains a major clinical challenge. Because the pathological results were difficult to obtain, the detection of radionecrosis still mainly relies on imaging. Radionecrosis shows annular enhancement on imaging, which is quite similar to a tumour. Therefore, it is not clear whether T1-enhanced lesions are related to real radionecrosis [11], [12].

There are several factors in the pathogenesis of RT-induced RBI, including vascular injury, white matter injury, demyelination, and axonal injury, among others. A study by Nagesh et al. [13] demonstrated that white matter injury was mainly caused by progressive dose-dependent demyelination, which mainly occurs within the first few months after RT in high-dose areas but takes longer to detect in low-dose areas. Since white matter injury is closely related to microvascular injury, ischaemia and microvascular hypoxia eventually lead to radionecrosis [14]. Therefore, early identification of acute microscopic changes in white matter caused by RT is vital for the accurate prediction and diagnosis of late RBI [15].

MRI provides qualitative and quantitative information for the evaluation of RBI. Due to microvascular injury, destruction of the blood–brain barrier, demyelination, and neuroinflammation in early radiation injuries in brain tumours [16], conventional MR can only obtain anatomical information and cannot accurately quantify and evaluate the response of white matter to radiation. Radiomics technology can reveal the outcome of diseases and normal tissues in diagnosis and treatment by mining the deep information in images [17]. Therefore, MR radiomics has important application potential in revealing microscopic changes in white matter and dynamically tracking the RT response, while different MR sequences could reflect different information about tissues. Although multisequence MR radiomics has a broader application potential, there is presently a lack of literature on such research.

In this study, it was observed that the features were mainly concentrated in the dose gradients 5–10 Gy, 20–30 Gy, and 30–40 Gy of multisequence MR images. A study by Connor et al. [18] confirmed that white matter injury could occur even after low-dose (5–10 Gy) RT. This was consistent with the results of this study, which showed that the number of significantly different features at the dose gradient of 5–10 Gy under the DWI sequence was the largest, and the rate of change of the features was the most significant when compared before and after RT. It was confirmed that the features of MR radiomics could reveal microscopic changes in white matter. Furthermore, although there was no general feature in the low-dose region (0–5 Gy), there was also a significant RT response in the ultrastructure of the white matter receiving low-dose radiation.

In this study, it was observed that there was no general feature under all dose gradients, the features T1 and T2FLAIR had a general feature under the low-dose gradients 5–10 Gy and 10–15 Gy, and the changes showed an upwards trend with an increase in the dose. There were 20 general features in T1+C under high-dose gradients of 20–30 Gy, 30–40 Gy, and 40–50 Gy. Except for the feature GLSZM-GreyLevelNonUniformityNormalized, the changes first decreased and then increased in a dose-dependent manner. This may be because the radiation-induced changes in the blood–brain barrier and vascular permeability increase the permeability of the contrast medium, thus improving the visibility of microscopic changes in white matter on T1+C^7^. Therefore, it was more advantageous to apply multisequence MR radiomics to predict or track the dynamic changes in white matter.

This study also analysed the radiomics features of DWI and ASL. A study by Melanie et al. [19] confirmed the relationship between microhemorrhage and white matter injury after RT. In the local region around the microhemorrhage focus, the fractional anisotropic changes in white matter decreased by 21.4% every year, which exceeded the fractional anisotropic change in naturally ageing white matter. ASL revealed changes in blood flow in the white matter, and the rate of change in the features before and after RT was the most significant (33.25%) at 30–40 Gy. This result was consistent with that of a previous study by our group, which analysed the relationship between perfusion changes before and after RT and RT doses [20]. According to the changes in radiomics features of ASL, it was suggested that the radiation dose for the normal brain region should be maintained below 30 Gy. Through the analysis of different MR sequences with the same dose gradient, this study demonstrated that the general features that changed significantly under the dose gradient of 30–40 Gy were T1 and T1+C, and the general features of different sequences were first-order category features, which indicated that such features tended to be stable under various sequences. Therefore, this feature should be preferred for analysis. Because of the influence of high signals of oedema in MR images, the T2 sequence was meaningless under dose gradients of 0–5 Gy and 40–50 Gy. Our present study did not find a more significant change pattern under the low-dose gradient of 0–5 Gy and high-dose gradient of 40–50 Gy.

In this study, it was observed that more in-depth anatomical and metabolic information could be obtained by the use of multisequence MR radiomics, which is a feasible method for the clinical monitoring of the changes in white matter caused by radiation. The main advantages are that all patients underwent standardized scanning, and previously used thin-layer (3 mm layer thickness, 0 mm layer interval) scanning images were adopted to track the changes in radiomics features of white matter. Compared to the traditional scanning method (slice thickness of 5 mm and interval of 2 mm), this method had higher accuracy and better response to the changes in white matter with RT. In this study, the time, space, and dosimetry model of radiation-induced injury could not be established. We intend to monitor the occurrence of radiation injury among patients in the future through follow-up. The correlation between the changes in radiomics features, radiation dose, and radiation white matter injury was analysed, and a predictive model of radiation-induced brain injury based on dose and radiomics features was established. This model can guide individualized RT and evaluate the risk of radiation-induced injury in patients [21].

In conclusion, the results of this study demonstrated that MR radiomics features can reveal the response of white matter to different doses of radiation. MR T1-enhanced images should be the first choice for radiomics feature extraction, which provides a feasible method for dynamic tracking of white matter radiotherapy response and early prediction of radiation damage.

## Data Availability

The datasets generated and/or analysed during the current study are not publicly available because they involve hospital patient information. However, they are available from the corresponding author upon reasonable request (chenming_53324@163.com).

## Abbreviations

RT: Radiotherapy
RBI: Radiation-induced brain injury
MRI: Magnetic resonance imaging
DTI: Diffusion tensor imaging
T2FLAIR: T2 fluid-attenuated inversion recovery sequence
DWI: Diffusion-weighted imaging
3D-ASL: Three-dimensional arterial spin labelling
CBF: Cerebral blood flow
OARs: Organs at risk
IMRT: Intensity-modulated radiotherapy
ROI: Regions of interest
